# Integrating Healthy Nutrition Standards and Practices Into Food Service Contracting in a Large US County Government

**DOI:** 10.5888/pcd21.230220

**Published:** 2024-03-07

**Authors:** Michelle Wood, Brenda Robles, Jacqueline Beltran, Tony Kuo

**Affiliations:** 1 Division of Chronic Disease and Injury Prevention, Los Angeles County Department of Public Health, Los Angeles, California; 2 Research Group on Statistics, Econometrics and Health, University of Girona, Girona, Spain; 3 Department of Health Policy and Management, Fielding School of Public Health, University of California, Los Angeles; 4 Department of Family Medicine, David Geffen School of Medicine, University of California, Los Angeles; 5 Department of Epidemiology, Fielding School of Public Health, University of California, Los Angeles; 6 Population Health Program, Clinical and Translational Science Institute, University of California, Los Angeles

## Abstract

**Purpose and Objectives:**

Although considered a promising model of practice, integrating healthy nutrition standards and practices into a large county government’s contracting process with food vendors has not been widely described in empirical literature. We conducted an implementation evaluation project to address this gap.

**Intervention Approach:**

County of Los Angeles food vendors provide food or meals annually to more than 100,000 employees and millions of clients and visitors. In 2011, the County of Los Angeles Board of Supervisors adopted a policy to integrate healthy nutrition standards and practices into its requests for proposals (RFPs) and contracting process with food vendors. The policy required all contracts awarded to adhere to these new standards.

**Evaluation Methods:**

In 2011, the Los Angeles County Department of Public Health (DPH) began reviewing RFPs for food services for county departments that procured, served, or sold food. From 2011 through 2021, DPH applied a 4-pronged formative–evaluative approach to help county departments implement the Board of Supervisors policy and ensure that nutritional requirements were appropriately integrated into all RFPs for new and renewing contracts with food vendors. We focused our evaluation on understanding the process and tracking the progress of this policy intervention. Our evaluation included 13 key informant interviews, a 2-part survey, reviews of contract data, and synthesis of lessons learned.

**Results:**

Based on reviews and subsequent actions taken on more than 20 RFPs, DPH successfully assisted 7 county departments to incorporate healthy nutrition standards and practices into their food vendor contracts. Implementation of the food policy encountered several challenges, including staffing and training constraints and a limited infrastructure. An iterative approach to program improvement facilitated the process.

**Implications for Public Health:**

Although the model for integrating healthy nutrition standards and practices into a government contracting process is promising, more work is needed to make it less resource-intensive and to increase user buy-in.

**Summary**

**What is already known about this topic?**

Integrating healthy nutrition standards and practices into the procurement process of an institutional food service is a promising public health strategy for improving nutrition.

**What is added by this report?**

We describe how a large county government operationalized a model practice to integrate nutritional requirements into its procurement process with food vendors.

**What are the implications for public health practice?**

Healthy nutrition standards and practices can change the quality of food served. Although implementation of such standards is feasible, organizational barriers exist across the various phases of the process.

## Introduction

Recent national data suggest that most of the US population has or is at risk of developing chronic diseases such as diabetes, hypertension, or heart disease (1). Data from regional population health surveys point to a similar pattern at the local level. In 2021, 33.5% of Los Angeles County adults reported ever being diagnosed with hypertension or prehypertension and 12.1% with diabetes (2). These and other chronic conditions represent a major public health problem that has substantial social and economic costs (3).

Diet plays a central and critical role in the development of chronic diseases (4,5). Diets high in sugar, sodium, saturated fat, and trans-fatty acids and the nutrients and ingredients in processed foods are linked to chronic ailments (6–8). The Dietary Guidelines for Americans (DGA) encourage increased intake of fruits and vegetables, whole grains, dairy foods, and lean proteins; they also encourage limited consumption of foods high in sodium, added sugars, and saturated fat (9). Since 2010, federal and local governments have increased efforts to integrate DGA-recommended nutrition standards and behavioral economics strategies into their food service contracting processes (10–12). At the federal level, nutrition standards are derived from DGA and form the foundation of the Food Service Guidelines for Federal Facilities of the US Department of Health and Human Services and the US General Services Administration. These guidelines are intended for government-operated food services and, as a model, highlight the importance of implementing standards in food service as a way to enhance population-level nutrition and public health (13). Although long considered an innovative and promising model of practice (14,15), limited literature has been published on the implementation of such food service requirements through institutional or governmental policy.

## Purpose and Objectives

The objective of our implementation evaluation was to address this gap in research and practice by describing how a large county government integrated healthy nutrition standards and practices into the food service contracting process of its departments. We present practice-based experiences and lessons learned from the County of Los Angeles in implementing such a policy, from 2011 through 2021. With the county’s extensive reach, which included over 100,000 employees and millions of annual clients and visitors, this decade-long food policy had the potential to generate significant health effects across the diverse communities it served.

## Intervention Approach

### Historical context

In response to the growing prevalence of obesity and related chronic diseases, particularly among its employees, in March 2011 the County of Los Angeles Board of Supervisors adopted an organizational policy on food quality entitled Healthy Food Promotion in LA County Food Services Contracts (16). This policy called for the Los Angeles County Department of Public Health (DPH) to ensure that healthy nutrition standards and practices were incorporated into all county food service and vending solicitations or requests for proposals across all the government’s 37 departments. DPH designed a review procedure to ensure that these nutrition requirements (eg, food purchasing and serving standards for fruit, vegetables, grains, protein, dairy, sodium, sugar) — including evidence-based behavioral economics strategies — were accurately incorporated and faithfully executed in contracts with food vendors (17).

### Present day action

The Board of Supervisors landmark policy remains active to this day. A food policy and procurement (FPP) team in DPH continues to provide support and serve as this implementation program’s subject matter expert and lead. The team is presently tasked with reviewing all food-related RFPs initiated under the county government’s umbrella; it can make recommendations on nutrition standards and practices and on how each department should conduct business with their food vendors (Figure).

**Figure:**
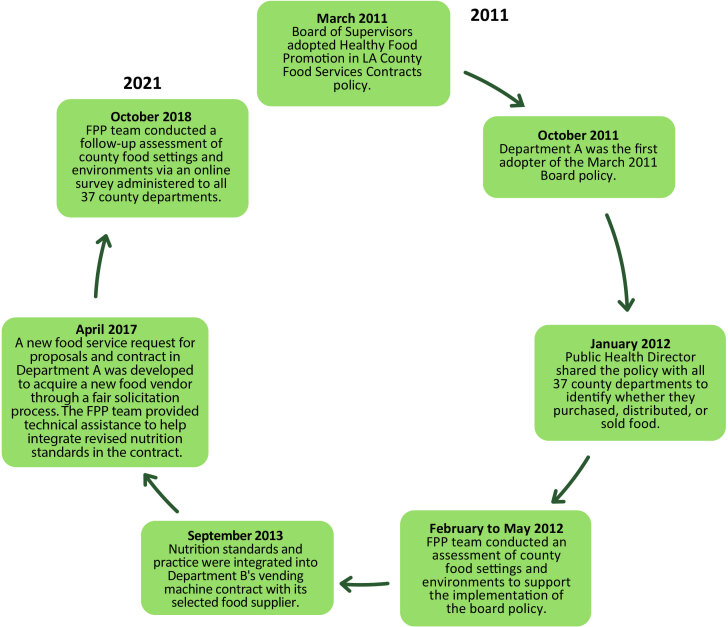
Timeline of the Los Angeles County Department of Public Health Food Policy and Procurement (FPP) team’s implementation process for reviewing and integrating nutrition standards and behavioral economics practices into more than 20 requests for proposals (RFPs) and food service contracts with food vendors conducting business with the County of Los Angeles, from 2011 to 2021. Department A refers to the first adopter of the March 2011 board policy. Department B refers to a second department that integrated nutrition standards and practices into its vending machine contract with its selected food supplier.

## Evaluation Methods

In our evaluation of the implementation of the Board of Supervisors policy, we sought to 1) summarize the contracting process that allowed the FPP team to integrate healthy nutrition standards and practices into the county’s RFPs for new and renewing food vendor contracts, 2) review and document contents of the reviewed RFPs and their contracts from 2011 through 2021, and 3) share practice-based experiences and lessons learned from the implementation of the board’s historic policy.

The FPP team applied a formative, multimethod, evaluative approach to achieve these goals and captured the progress and nuances of implementing the food policy. This approach consisted of 1) an organizational landscape analysis through key informant interviews, 2) a 2-part survey of county departments, 3) a document review of county food vendor RFPs and resulting contracts, and 4) a synthesis of lessons learned from implementing the policy. As an example, in 2012 (after the board’s policy was adopted but before the policy was implemented), the FPP team conducted an organizational landscape analysis with key informants to identify and better understand the types of contracts covered under the Board of Supervisors policy. This assessment included disseminating a memorandum requiring each of the 37 county departments affected by the policy to inform DPH of whether they operated any food services. “Food vendor” was defined as any vendor who prepares, sells, distributes, or serves food for the county. Key informant interviews were then conducted with representatives from departments that reported they purchased, distributed, or sold food.

To gauge implementation progress, in 2018 the FPP team administered a 2-part survey. The first part was emailed to selected participants from the county’s 37 departments. The second part was administered only to departments that indicated they operated food service venues or programs. A document review of county food vendor RFPs and their contracts, including a case assessment of an early county department adopter, was carried out to learn about the implementation process. Finally, a synthesis of lessons learned from the field was compiled to inform future policy implementation and refinements.

### Organizational landscape analysis via key informant interviews, 2012

We conducted a landscape analysis to 1) support the implementation of the Board of Supervisors policy, 2) engage key stakeholders to better understand the county’s diverse food environments (where food was prepared, served, or sold), and 3) inform the resources and planning needed to comply with the food policy. County departments were initially sent a memorandum asking them to complete a brief questionnaire indicating whether their department purchased, distributed, or sold food. Departments that indicated they did were then asked to designate a department contact who could participate in a key informant telephone interview with the FPP team. The team then scheduled interviews with the designated department contacts.

Interviews were carried out by using an interview guide informed by data and information from the literature (what was known about food policy adoption and implementation at the time) (15,17). The goal of the interviews was to gather information from county departments before implementation of the Board of Supervisors policy. Information gathered consisted of types of food venues and populations served by each of the departments; current contracts, such as the number of agreements and food vendors each department was handling; the existing nutrition guidelines or practices the department followed; the number of meals the department prepared, sold, or served across their food venues and environments; and any challenges or barriers encountered with purchasing or preparing healthy food items. Each interview took about 1 hour.

### Two-part survey, 2018

The FPP team developed the 2-part survey in 2018 and sent it to all 37 county departments. The survey’s primary goal was to better understand the departments’ approach to conducting business with their food vendors and to identify any major changes that may have occurred in their approach since the adoption of the 2011 Board of Supervisors policy. The first part of the survey was a brief questionnaire designed to collect information on 1) each department’s name, number of employees, and the physical and mailing addresses for their headquarters; 2) the name and contact information of the staff member(s) who would represent each department and complete the survey; and 3) whether each department at the time of the survey distributed, sold, or served food or beverages to county employees, their dependents, or visitors. The questionnaire was web-based and programmed by using the SurveyMonkey platform (SurveyMonkey). The first part of the survey was emailed to all 37 departments, asking the appropriate contact or representative to complete the questionnaire. The second part was sent to departments that indicated they prepared, sold, or served food or beverages at their facilities to get further details about their food service environments (eg, types of venues, types of contracts, populations served, food service operation characteristics).

Data and information obtained from interviews and the survey were reviewed and synthesized as lessons learned or field-tested practices (Table 1). These results are currently being used to update and refine policy implementation efforts.

**Table 1:** Policy^a^ to Integrate Healthy Nutrition Standards and Practices Into Requests for Proposals^b^ (RFPs) for Contracting with Food Vendors, County of Los Angeles Departments, by Food Setting and Environment, 2011–2021

Type of food service setting or food environment	Food service site or venue	Audience	How many RFPs, including the proposed scope of work of prospective food vendors, were reviewed by DPH during the contracting process	Estimated number of people reached or exposed to a program or intervention per year^c^
**Meal distribution programs**	Parks and recreation sites that administer the Summer Food Service and After School Snack programs	Children and adolescents	1 RFP reviewed	88,391
	Shelter care center within a court	Children and adolescents in county custody awaiting court hearings	2 RFPs reviewed	9,612
**Detention facility meal programs**	Juvenile halls and camps	Adolescents and young adults in detention facilities	6 RFPs reviewed	2,714
**Concession stands**	Beach concession stands and mobile carts	Community members and visitors	1 RFP reviewed	550,000
**Worksite cafeterias**	Workplace cafeteria (Department A^d^)	Employees and visitors	2 RFPs reviewed	1,400
	Workplace cafeteria	Employees and visitors	1 RFP reviewed	2,334
	Public hospital cafeterias, snack shops, and vending machines	Employees, patients, and visitors	3 RFPs reviewed	393,321
	Workplace cafeteria	Employees and visitors	1 RFP reviewed	1,465
	Health center cafeteria	Employees, patients, and visitors	2 RFPs reviewed	32,380
**Restaurants**	Restaurant on government beach property	Community members and visitors	1 RFP reviewed	34,790
**Vending machines**	Worksite locations (Department B^e^)	Employees and visitors	1 RFP reviewed	82,516
	Recreation facilities	Employees, community members, and visitors		15,074

Abbreviation: DPH, Los Angeles County Department of Public Health.

a Healthy Food Promotion in LA County Food Services Contracts policy (16).

b The Board of Supervisors policy (16) requires the review of RFPs to integrate healthy nutrition standards. In some cases, county departments used other types of solicitation mechanisms (eg, invitations for bids, *requests for statements of interest*). Each row represents a different county department or food service type with separate RFPs and contracts.

c Population reach estimates were based on publicly available data or internal records and determined by using the definitions and methodology described in Robles et al (18).

d County department selected as a case example of the policy implementation in the study.

e County department that administers a large vending machine contract.

### Review of food vendor requests for proposals

With results from the key informant interviews, and subsequent findings from the 2-part survey, the FPP team developed a document review procedure to streamline the process of examining each food service RFP. These results helped the team plan for and update the overall review process, by 1) categorizing the types of food venues operating in the county, 2) gaining a better understanding of the population served by each contract, 3) determining whether a department was using existing nutrition standards, 4) documenting the implementation challenges each department encountered, and 5) learning how best to build policy implementation capacity (eg, tailored technical assistance, support tools) across the county. The review procedure has been refined iteratively over the past 10 years to improve the implementation of the Board of Supervisors policy.

Since the policy’s adoption in 2011, the FPP team has reviewed and paved the way for 7 county departments to incorporate healthy nutrition standards and practices into 21 food service RFPs; most of the resulting contracts were successfully executed. For each RFP, the review focused on adherence to the county’s recommended nutrition standards and practices and the factors that may have facilitated or impeded the execution of these requirements (Table 2). These factors included vendor readiness, the feasibility of standardizing nutrition quality, types of contracts and timelines (ie, new or renewing), and types of food service settings or environments encountered (ie, distributive meal programs, institutionalized meal programs, concession stands, worksite cafeterias, restaurants, or vending machines). The review also provided an opportunity for the FPP team to make recommendations regarding contract language that focused on facilitating the feasible integration and operationalization of healthy nutrition standards and practices. In most cases, departments accepted the FPP team’s recommendations.

**Table 2:** Nutrition Standards and Practices^a^ Integrated Into Department A’s^b^ Request for Proposals (RFPs) in 2017, Policy to Integrate Healthy Nutrition Standards and Practices Into Food Vendor Contracts, County of Los Angeles, 2011–2021

Aspect of food service	Requirements for vendor proposals in response to RFPs^a^
**Proposal preparation and submission of the Work Plan**	
Menu	Description of menu options including but not limited to breakfast menu selections, value menu, lunch entree selections, healthy food choices, snacks, and beverages. Please note that the successful contractor is required to comply with the Concession Nutrition Standards identified in Part II, Exhibit H^c^. Description should also include: i. A list of prices as well as nutritional information for all menu options. ii. More than two healthy low fat and low calorie selections for lunch. iii. Portion sizing iv. Quality of food v. Indication that menu items will comply with Concession Nutrition Standards identified in Part II, Exhibit H^c^.
Vending machine operations	A description of vending machine services, qualifications, experience, staffing, and schedules . . . Please note that the successful contractor shall be required to comply with the County of Los Angeles Vending Machine Nutrition Policy, 3.115, as described in Exhibit G^d^.
Wellness and sustainability policy	A description of the Proposer's existing wellness and sustainability policy that demonstrates their commitment to promoting wellness programs such as healthier menu offerings and beverages, menu labeling, healthy checkout registers, etc. to support healthy eating. The description of the wellness and sustainability policy should also demonstrate how this policy has been implemented.
Registered dietician	The Proposer's staffing plan must include a Registered Dietitian who will provide menu and meal planning services that comply with the Concession Nutrition Standards, Exhibit H^c^ and as needed to confer with Department of Public Health to implement the standards.
**Sample agreement on the scope of work for cafeteria and vending machine services**	
Concession nutrition standards	Plan and implement menus that contain healthy food and beverage choices as defined in Exhibit H^c^, Concession Nutrition Standards.
	Contractor shall submit a nutritional analysis of all menu items to the DPH and the Contract Manager at the commencement of the Contract and when menus change with the introduction or modification of new menu items to confirm adherence with all nutrition standards in this Contract. Please refer to Exhibit M^e^ for a Sample Nutritional Analysis . . . Food Production and Sales Record.
Sodium reduction	Implement the DPH’s Sodium Reduction Plan within 12 months of this Contract's commencement. The Contractor shall work with DPH staff, as well as the Contract Manager to comply with the sodium standards for purchased food categories. The DPH Sodium Reduction Plan is attached as Exhibit L^f^ to this Contract.
Menu labeling	Prepare and provide weekly menus, which include prices and a description of each item. Contractor shall distribute menus on Thursday afternoon for the following Monday service. The menu shall also list the nutritional information for each item in accordance with the Federal menu labeling requirements set forth under the Patient Protection and Affordable Care Act of 2010 in Exhibit K^g^, Menu Labeling Requirements. This requirement shall also apply to all future menus or proposed changes.
Signage	Clearly indicate healthy menu items. Contractor shall add symbols to the menu to identify items that feature vegetarian or vegan menu items (when applicable). Contractor shall also add symbols to the menu to identify menu items that feature local produce.
	In consultation with the County Contract Manager and DPH, prominently display *Choose Health LA* h signage (signage shall be provided by DPH) that promotes healthy food and beverage options made available by the Contractor. Signage indicating availability of fresh, cold tap water at no charge shall be placed at fountain drink machine or hydration station. Signage identifying reduced-size portion entree options and combination meals with the alternative option to select bottled water and a nonstarchy vegetable or fruit as a side item shall be displayed.
Product placement	Position healthy food and beverage items prominently in the cafeteria with easy access for customers.
	Contractor shall only display food and beverage items meeting Concession Nutrition Standards (Exhibit H^c^). • Healthy snacks and water shall be placed within 5 feet of all checkout registers. • Candy bars, cookies, chips, and sugar-sweetened beverages (sugar-sweetened beverages include all sodas, fruit drinks, sport drinks, low-calorie drinks, and other beverages that contain added caloric sweeteners, such as sweetened tea, rice drinks, bean beverages, sugarcane beverages, and nonalcoholic wines) shall be removed from checkout register area or at point-of-purchase. • Fresh fruit shall be displayed within reach of the checkout register. • Only healthy beverages, as defined in Exhibit H^c^, shall be displayed in eye-level sections of beverage cases. • Only healthy snacks/desserts, as defined in Exhibit H^c^, shall be displayed in eye-level sections of display areas. • Healthy food entrees and side items, as defined in Exhibit H^c^, shall be placed at the front of each food service area.
Pricing incentives	Prices of healthy entrees, side items, snacks/desserts, and beverages, as defined in Exhibit H^c^, Concession Nutrition Standards, shall not exceed the price of other menu options . . .
	Pricing for the salad bar and pre-packaged salads shall be competitive with other food entree options.
Catering	Catering menus shall comply with the Concession Nutrition Standards set forth in Exhibit H^c^.
Vending machines	Comply with the County of Los Angeles Vending Machine Nutrition Policy, 3.115, as described in Exhibit G^d^.
Monitoring adherence to nutrition standards	Comply with all nutrition guidelines outlined in this Contract, as well as any future Board of Supervisors’ policies concerning nutrition guidelines. The nutritional guidelines may be revised periodically to ensure they meet current dietary recommendations. County will provide the Contractor with the revised nutritional guidelines as they become available. DPH may periodically monitor the Contract to ensure the Contractor is in compliance with Exhibit H^c^, Concession Nutrition Standards. Contractor is required to maintain and submit quarterly to the County upon the Contract Manager's request, the following records: food production records, itemized monthly sales, and a complete nutrition analysis of all menu products/items offered. Please refer to Exhibit M^e^ for a Sample Nutritional Analysis Food Production and Sales Record. DPH shall review records and communicate its findings to the Contract Manager. Failure to comply with the Concession Nutrition Standards may, in the County’s sole discretion, constitute a breach of this Contract.
Registered dietician	Contractor shall provide services from a registered dietitian as necessary to provide menu and meal planning services that comply with the Concession Nutrition Standards, Exhibit H^c^, and as needed to confer with DPH to implement the standards. The Contractor shall immediately notify the Contract Manager of any change of the registered dietitian.

Abbreviation: DPH, Los Angeles County Department of Public Health.

a Excerpted from the County of Los Angeles Department of Public Works Request for Proposals for Cafeteria and Vending Machine Services at the Department of Public Works Headquarters (2017-PA011) to illustrate what nutrition standards were integrated into the RFP to implement requirements of the Board of Supervisor’s Policy. These standards were revised in 2020.

b County department selected as a case example of the implementation of County of Los Angeles Board of Supervisors policy, Healthy Food Promotion in LA County Food Services Contracts (16).

c Exhibit H: Los Angeles County Department of Public Health Concession Nutrition Standards. The standards set nutrition limits for snacks/desserts, main dish/entree, side items, beverages, combination meals, condiments, fruit, vegetables, grains, protein, and dairy. They include standards for food preparation methods, local produce, and behavioral economics strategies (ie, product placement, menu labeling, signage, price incentives).

d Exhibit G: County of Los Angeles Vending Machine Nutrition Policy. The policy sets nutrition guidelines for snacks and beverages sold in County of Los Angeles vending machines.

e Exhibit M: Sample Nutritional Analysis Food Production and Sales Record. This exhibit presents samples of data sources for menus, nutritional information, food production, and sales records. These data sources support the assessment of contract terms related to the implementation of nutrition standards.

f Exhibit L: Implementing a Sodium Reduction Plan. This exhibit specifies a plan to implement purchasing standards for the sodium content of food products.

g Exhibit K: Menu Labeling Requirements. This exhibit specifies menu labeling requirements including the display of calories for all food items.

h Choose Health LA is an educational campaign directed at county employees and the community to promote healthy eating through informational materials such as signage, table tents, and an informational website at worksite cafeterias in county buildings.

The implementation of healthy nutrition standards and practices has evolved over the past 10 years, especially for environments like cafeterias, cafés, and concessions. The integration of these standards has become more venue-specific and often requires careful consideration of where the food is being sold or served. Making this distinction is important because where the food is being sold (cafés, regular food stands) may be quite different from where meals are being served (eg, county’s detention facilities, no-cost or reduced-cost meal programs). Based on these lessons learned, and the latest nutrition science, in 2020 the FPP team revised its DPH Nutrition Standards for Prepared Foods, Snacks, and Beverages.

### Documentation and synthesis of lessons learned

Ten years of lessons learned from the various RFPs, such as what facilitated the policy implementation and the challenges encountered during implementation, were documented and synthesized by the FPP team. These were used to build a comprehensive compendium (inventory) of the strategies used to integrate healthy nutrition standards and practices into the county’s food vendor contracting process. A series of brainstorming meetings based on the FPP team’s implementation and evaluation findings were convened internally by the team throughout the policy implementation period to develop, establish, and periodically refine the policy objectives, nutrition and practice recommendations, and the review procedure that aided the processing of the RFPs.

## Results

### Organizational landscape and the two-part survey

In 2012, for the organizational landscape, 28 (76%) of the 37 county departments responded to the initial DPH memorandum requesting information from departments on whether they purchased, distributed, or sold food. Thirteen departments that indicated in their response that they purchased, distributed, or sold food were subsequently interviewed.

Twenty-six (70%) of the county departments participated in the first part of the 2018 survey. A total of 34 representatives, 1 to 2 per department, completed the questionnaire. More than half (53.0%) of the respondents were identified as being an administrative deputy or an administrative or section manager. Of these 26 departments that participated in the first part of the survey, only 15 identified as distributing, selling, or serving food to county employees, dependent populations, or visitors. All 15 departments completed the questionnaire. 

Results of the second part of the survey indicated that each department spent an average of $2,808,340 per year on food and beverages; the total annual spending across all departments ranged from $500 to $27,000,000. Most (66.7%) indicated they purchased food and beverages internally for department-sponsored meetings and events. About half (53.3%) reported they offered food at no or low cost to community members and to those who depended on food programs such as CalFresh (Supplemental Nutrition Assistance Program) and the National School Lunch Program. Less than a third (25.6%) reported they operated their own food service (ie, via their own department staff). More than half (53.3%) reported contracting most of their food services needs to external companies such as Aramark, Morrison Healthcare, or Sodexo. Although less than half (46.7%) indicated their department offered healthy foods (eg, fruit, vegetables, whole grains, minimally processed foods), about 60% had a registered dietician on staff who either worked for the department or for the contracted food vendor.

### Document review and synthesis of lessons learned

Based on reviews of more than 20 food-related RFPs issued from 2011 through 2021, the FPP team drew several takeaways and lessons learned from the county’s effort to implement the Board of Supervisors policy. For example, the reviews provided information and pointed to key places in the county’s RFP and contracting process where standards and best practices could be reasonably incorporated without leading to costly delays or issues with execution of the contracts. The resulting 4-phase contracting framework provided the FPP team with a roadmap to guide implementation decisions, inform strategy selections, provide an illustrative case example, and discuss implementation facilitators and challenges.

### The 4-phase food service RFP and resulting contracting process

The addition or integration of evidence-based nutrition standards and practices into the county’s food service RFP and contracting process for food vendors was lengthier and more resource-intensive than originally anticipated. For example, the FPP team reviewed more than 20 county RFPs and related documents on food services and food procurement at the beginning of policy implementation and continued to do so throughout the 10 years to keep pace with new and renewing contract development. For renewals, many contracts had expired and RFPs were subsequently reissued after the board policy was adopted. The RFP process across all departments turned out to be less uniform than initially anticipated, requiring additional time to gain a better understanding of the differences and similarities between departments. For instance, government food programs operated by the County of Los Angeles are often governed by existing local, state, or federal laws (eg, Title 15, National School Lunch Program). Adding new standards and practices required careful review of these existing laws so that the new Board of Supervisors policy did not disrupt, contradict, or exceed existing legal requirements.

To develop the framework on contracting (Table 3), the FPP team worked collaboratively with at least 7 county departments to document and understand the nuanced workflows that governed the various RFPs. The FPP team used this information to conceptualize the county’s food service contracting process as a 4-phase solicitation and contract execution procedure. This procedure incorporated healthy nutrition standards and practices strategically at places where they could be inserted or implemented along the continuum of actions, taking into account each department’s needs and considerations (eg, program readiness, laws a department had to follow, how large or small food vendors worked with each department). The 4 phases are as follows: 1) the development of the RFP (an optimal time for including standards and practices as part of the proposed scope of work for each contract), 2) the release of the RFP (an opportune time to educate prospective vendors about the required nutrition standards and practices), 3) the evaluation of vendor proposals (an important leverage point where information about complying with the Board of Supervisors policy could be emphasized), and 4) the selection of the food vendor (timepoint where the final execution of the contract allowed for the codification of the healthy nutrition standards and practices, that is, standards and practices that could be required in the food vendor’s contractual agreement).

**Table 3:** Four Phases^a^ of Food Service Contracting, Policy^b^ to Integrate Healthy Nutrition Standards and Practices Into Requests for Proposals^c^ for Contracting with Food Vendors, County of Los Angeles, 2011–2021

Phase 1	Phase 2	Phase 3	Phase 4
Develop an RFP that integrates healthy nutrition standards and practices into the contract(s) of food vendor(s) • DPH meets with a county department to discuss the current food environment, existing regulations that may govern food quality and the RFP process • DPH reviews the draft RFP and develops recommended nutrition standards, and behavioral economics strategies if applicable, for inclusion in the RFP • Nutrition standards are presented to the county department, and mutually agreed upon standards are finalized • Contract language describing nutrition standards are developed and integrated into the final RFP (eg, scope of work, minimum mandatory requirements)	Release the RFP with the nutrition standards and practices • County department releases the RFP with nutrition standards and behavioral economics strategies if applicable • Proposers (food service operators) may develop and submit written questions regarding the RFP • The county department that released the RFP develops a list of questions and answers, which is shared with the public • Food vendors are required to participate in a mandatory proposer’s conference (eg, facility walk-through). DPH participates, if appropriate, at the mandatory proposer’s conference and provides an overview of the nutritional standards • Proposals are submitted to the county department	Evaluation of food vendor proposals • An evaluation committee is developed by the county department to evaluate and score the proposals based on set criteria, including compliance to nutrition standards • Food vendors must meet minimum mandatory requirements of the RFP • Food vendors are evaluated on criteria such as experience, background, references, and their proposal to meet work plan requirements including healthy nutrition standards	Food vendor selected • Department selects new food vendor and enters into contract negotiations • The new vendor and contract is submitted to the County Board of Supervisors for approval • Final contract awarded by County of Los Angeles Board of Supervisors to selected food service operator

Abbreviations: DPH, Los Angeles County Department of Public Health; RFP, request for proposal.

a Entry points where integration of healthy nutrition standards and practices could be accomplished or strengthened.

b Healthy Food Promotion in LA County Food Services Contracts policy (16).

c In some cases, county departments used other types of solicitation mechanisms (eg, invitations for bids, *requests for statements of interest*). There may also be some differences in the wording, and requirements of RFPs across county departments and food service settings.

On average, each contract review — activities such as reviewing the RFP, developing nutrition standards and contract language, corresponding with county departments — took approximately 2 to 3 weeks. In the early stages of policy implementation, reviews took much longer, because the implementation program was new and the FPP team was still building the program’s infrastructure. Reviews of RFPs for low- or no-cost meal programs, and food served to populations that are dependent on meals, such as those in detention facilities, often required additional time and extensive review. This was due to the need to ensure that the proposed standards and practices adhered to requirements set by local, state, and federal laws.

### Illustrative case example

The contract for cafeteria and vending machine vendors of the first county department to adopt the board’s policy, referred to in this article as Department A, was an example of how the FPP team worked with one of the county’s largest departments to introduce and embed healthy nutrition standards and practices into their food service RFP and resulting contract. Department A’s on-site cafeteria sold food to county employees and visitors at their department headquarters. Their RFP was the first solicitation the FPP team worked on; the team’s review took place shortly after the enactment of the Board of Supervisors policy. As the first RFP to be reviewed, the sequence of steps the FPP team took to move the process forward was iterative, and a learning experience. The workflow required the development of an entirely new set of contract language that delineated required nutrient standards and limits to follow as well as the “dos and don’ts” of practices for purchasing food under these new standards. Language specified how signage and behavioral economics strategies should be used in the cafeteria. Some of the recommended standards and practices were menu labeling, requiring at least 2 healthy entrée selections and 2 healthy side options on the menu, using pricing incentives, specifying nutritional requirements for combination meals, developing and implementing a sodium reduction plan, setting size limits for sugar-sweetened beverages, and requiring 25% of snack options to be healthy (ie, low in sugar and sodium). Many of the standards and practices were also applied to vending machines; a separate nutrition policy, the County of Los Angeles Vending Machine Nutrition Policy (19), guides nutritional quality of foods in vending machines.

A half-decade after the initial contract was developed, Department A’s cafeteria and vending machine services contract expired, and a new RFP cycle was initiated in 2017. This new cycle gave the FPP team an opportunity to apply lessons learned from the first contracting process to streamline and improve the second RFP (Table 2). For example, a major challenge with Department A’s RFP development was the need to draft contract language where allowable nutrient limits were clearly delineated and could be easily enforced, but at the same time, flexible enough to account for unanticipated problems in putting the program into practice. Standards had to address clientele dissatisfaction with the food or a decline in sales volume as a result of changes in food options, account for supply chain and workflow issues that were barriers for food vendors seeking to comply with the recommended standards and practices and consider potentially higher costs of food low in sodium or sugar. Sorting through many of these challenges was an invaluable learning experience for the FPP team. Department A’s experience built the FPP team’s confidence and gave it the opportunity to experiment with the review procedure and with the standards and practices that were ultimately recommended to improve the nutritional quality of foods offered by selected food vendors from each of the RFPs. In making these nutritional recommendations, we synthesized and applied lessons learned from integrating nutritional requirements into Department A’s and other county departments’ contractual processes with food vendors (Table 4).

**Table 4:** Facilitators and Challenges to Operationalizing the Board of Supervisors Policy^a^ to Integrate Healthy Nutrition Standards and Practices into Requests for Proposals for Contracting with Food Vendors, County of Los Angeles, 2011–2021

Facilitator	Description
Board policy institutionalized a contract review process	The board policy institutionalized a process to include nutrition guidelines as a standard of practice within the food contracting process.
Understanding the contracting process	Learning and understanding the contract solicitation process (ie, primarily for the RFP mechanism) of each county department that serves, sells, or distributes food was instrumental to policy implementation.
Stakeholder engagement	Building partnerships with county administrators and contract managers who oversee food contracts.
Tailored nutrition guidelines	Developing nutrition guidelines that can be integrated into all food contracts and venue-specific standards that meet the needs of specific food venues and target populations.
Multipronged approach to implementation	Implementing nutrition standards and other contract requirements requires technical assistance resources such as marketing, culinary training, and leveraging data to drive nutritional changes.
Resources to support implementation	Implementation resources and promotional materials are needed to educate implementers (eg, county employees, food vendors) and community members about nutritional changes.
Monitoring and evaluation of board policy	The FPP team provided ongoing monitoring and evaluation support to county departments throughout the 10-year period.
**Challenges**	**Description**
Complexity of contracting system	The contracting system is complex with various food service types and venues, departmental staffing, different contracts and grant units and workflows, operating procedures, and contract solicitation mechanisms. The contracting system across departments lacks structure and standardization.
Implementation capacity of smaller food vendors	Smaller vendors have less capacity (resources and staffing) to implement nutrition standards in contracts.
Lack of staff nutrition knowledge	County departments have limited staff capacity, training, and nutrition knowledge to implement nutrition standards.
Limited staffing for county-wide implementation	There is only one full-time–equivalent position dedicated to overseeing implementation of the board policy.
Challenges with contract monitoring and data collection	Limited resources for oversight and monitoring of food contracts for adherence to contract terms and nutrition standards. Challenges with collecting nutrition analysis and procurement data from county departments to identify opportunities for replacing unhealthy products with healthier alternatives.

Abbreviation: FFP team, Food Policy and Procurement team within the Los Angeles County Department of Public Health.

a Healthy Food Promotion in LA County Food Services Contracts policy (16).

### Lessons learned: facilitators

The Board of Supervisors policy played a pivotal role in prioritizing and institutionalizing nutritional quality in the food service contracting process. The policy established a mindset that access to healthy food should be the norm in practice. The policy initiated a process in which DPH’s review of food-related RFPs and their contracts became an accepted routine practice, standardizing how the county conducts business with food vendors. Early in the implementation process and shortly after the policy’s adoption, efforts were made to understand the solicitation process of each county department. This understanding acknowledged that departmental protocols might differ, and that certain department staff and food vendors might need to be educated about the RFPs and contracts. Key insights from this phase guided the FPP team as they mapped the necessary implementation steps to translate the board’s policy into practice.

In the early phases of working with RFPs, the FPP team quickly recognized the importance of establishing strong partnerships with key members of county departments. These partnerships proved essential for effectively incorporating the recommended standards and practices into contract solicitations. Recognizing this, the team made a top priority of working diligently and respectfully with administrators overseeing a department’s food service contracts or the contract managers who monitored food-related contracts. The team also discovered that standards and practices had to align with each department’s overall business goals. These considerations reflected important realities that influenced the speed, or lack thereof, at which a given RFP could be developed and administered. After 10 years of policy implementation, the integration of healthy nutrition standards and practices into government food service RFPs and their resulting contracts has become a qualified success. However, investments in staffing and support were limited at the policy’s launch and remain underresourced to this day.

Implementing contract requirements involves a multipronged approach and technical assistance resources, such as culinary training, marketing, and leveraging data to drive nutritional changes. A robust evaluation component was imperative to guide and refine implementation. For instance, the FPP team provided ongoing monitoring and evaluation support to county departments throughout the 10 years; this was a key facilitator for implementing the food policy. Food environment assessments were conducted before and after contract execution for selected departments by using internally developed checklist tools that assessed the implementation of the recommended standards and practices (eg, implementation of behavioral economics strategies). Quality assurance reports with checklist results were developed for use by departments. The FPP team also developed implementation guides and resources (eg, toolkits, recipes) and nutrition promotional materials (eg, signage, table tents, decals) for staff and visitors to use. These resources played a critical role in educating implementers (county departments and food vendors), county employees, and community members who visited county food settings about the changes that were being made to the food at county facilities.

### Lessons learned: challenges

We encountered several notable challenges to integrating healthy nutrition standards and practices into the county contracting process. First, the contracting system across the county was complex and continues to pose challenges to this day. For instance, the learning curve for the FPP team was steep. It took the team some time to grasp the nuances of how contracts with food vendors functioned in the county. The county contracting process is also multilevel and nonlinear, with variations based on food service type or venue, department staffing, and inter- and intradepartmental workflow. Other challenges resulted from the diverse procedures among departments. Often, each department had separate contracts and grant units, each with its own operating procedures and solicitation mechanisms (eg, RFPs, invitations for bids, *requests for statements of interest*). The county system’s size and complexity may also explain why some departments were not involved in implementation, because they were unfamiliar with the board’s policy or had food vendor contracts that were signed for a long term (≥10 y), precluding meaningful review. Staff turnover and gaps in staffing may have precluded engagement, participation, and intracounty collaboration.

A second challenge was that some of the department administrative structures that were created to ensure checks and balances and a seamless pathway to contracting did not always work smoothly. Instead, they often generated operational inefficiency, which led to unforeseen delays with the RFP process and poor contract execution. For instance, the limited adherence to a strict timeline and a lack of standardization of the contracting process across several departments meant that even simple adjustments to contract language was a daunting task to coordinate, especially when these adjustments affected more than one department. Long delays became an undesirable norm because the sparse standardization and lack of structure frequently allowed for competing institutional priorities to redirect human resources away from the contracting process. In some instances, the FPP team never received final copies of the contracts or were not informed by the county department whether the contracts had been executed. In some cases, it was unknown or unclear whether the RFPs the FPP team worked on were ever released to the public.

Third, most contract managers within the county departments had limited nutrition knowledge and experience with operationalizing nutrition standards or implementing behavioral economics strategies. As such, the time required to train contract managers (eg, how to market healthier meals, how to improve consumer buy-in, how to collect relevant evaluation data) was a labor-intensive task. This was not something the FPP team, a small group with only one full-time–equivalent staff position dedicated to the policy’s implementation, had anticipated. Currently, the FPP team still has only one full-time–equivalent position allocated for implementing the Board of Supervisors policy. Other difficult-to-overcome challenges in implementation included cost and budget constraints, limited contract oversight, and limited institutional capacity to monitor contracts and activities for adherence to DPH-recommended nutrition standards and practices (17).

Lastly, the collection of data on food purchased, sold, and served by county departments posed multiple challenges. Often, the only pertinent data came from publicly available menus. Almost no nutritional analysis data were collected or reported by contracted food vendors, and in several cases, vendors did not use standardized recipes.

## Implications for Public Health

Although the Board of Supervisors policy adopted in 2011 has made tremendous progress in helping to solidify a review procedure and a set of nutrition recommendations for the county’s food service contracting process, notable challenges remain. Funding and human resources for ongoing administration, compliance monitoring, and program evaluation remain elusive, despite their being essential for sustaining implementation of the Board of Supervisors policy. Because of challenges with data collection and limited funding and human resources, the FPP team has been unable to assess the level of adherence to nutrition standards and practices in food services operated by the county. Most county-contracted food vendors shared only menus and have been unable to provide additional information on the nutrients and ingredients they use in food preparation. As a result, it has been difficult to fully assess whether healthy menus actually complied with the DPH-recommended nutrition standards and practices.

In summary, our model to integrate nutritional requirements into the county contracting process with food vendors appears to be a promising approach for institutionalizing healthy nutrition standards and practices in a large California county government. The approach’s goal was to increase the quality of food that government entities purchase, sell, and serve. Based on field findings and early evaluation results, our model has the potential to perform similarly in other agencies or jurisdictions interested in taking similar action within their organization to improve food quality, health, and financial sustainability (11,20–22).

Although some best practices introduced by the model require further research for codification, efforts on the ground and in the field should continue to build a business case for implementing food policies like the one embedded in the contracting process of the County of Los Angeles government. For instance, convening contracts and grants specialists from each of the relevant departments would be beneficial to discuss lessons learned and gather input on how best to leverage their department’s contracting process, enforcement mechanisms, and purchasing power to improve the nutritional quality of foods served or sold in county facilities.
